# A Systematic Approach to the Evaluation of the Coronary Microcirculation Using Bolus Thermodilution: CATH CMD

**DOI:** 10.1016/j.jscai.2024.101934

**Published:** 2024-04-03

**Authors:** Carlos Collet, Andy Yong, Daniel Munhoz, Takashi Akasaka, Colin Berry, John E.A. Blair, Damien Collison, Thomas Engstrøm, Javier Escaned, William F. Fearon, Tom Ford, Tommaso Gori, Bon-Kwon Koo, Adrian F Low, Steve Miner, Martin K.C. Ng, Takuya Mizukami, Hiroki Shimokawa, Nathaniel R. Smilowitz, Nadia R. Sutton, Johan Svanerud, Jennifer A. Tremmel, Takayuki Warisawa, Nick E.J. West, Ziad A. Ali

**Affiliations:** a Cardiovascular Center Aalst, OLV Clinic, Aalst, Belgium; b Concord Repatriation General Hospital, University of Sydney, New South Wales, Australia; c Department of Advanced Biomedical Sciences, University Federico II, Naples, Italy; d Department of Cardiovascular Medicine, Wakayama Medical University, Wakayama, Japan; e School Cardiovascular and Metabolic Health, University of Glasgow, Glasgow, United Kingdom; f Department of Medicine, University of Chicago Medicine, Chicago, Illinois; g Golden Jubilee National Hospital, Clydebank, Glasgow, United Kingdom; h Rigshospitalet, University of Copenhagen, Denmark; i Hospital Clinico San Carlos IDISSC, CIBER-CV and Complutense University of Madrid, Madrid, Spain; j Division of Cardiovascular Medicine and Stanford Cardiovascular Institute, Stanford University School of Medicine and VA Palo Alto Health Care System, Palo Alto, California; k Faculty of Health and Medicine, The University of Newcastle, Newcastle, New South Wales, Australia; l Department of Cardiology, University Medical Center and DZHK Partner site Rhein-Main, Mainz, Germany; m Department of Internal Medicine, Cardiology Centre, Seoul National University Hospital, Seoul, South Korea; n National University Heart Centre, Singapore; o Division of Cardiology, Southlake Regional Health Centre, Newmarket, Ontario, Canada; p Department of Cardiology, Royal Prince Alfred Hospital, Camperdown, Australia; q Division of Cardiovascular Medicine, Tohoku University Graduate School of Medicine, Seiryo-machi, Aoba-ku, Sendai, Miyagi, Japan; r Leon H. Charney Division of Cardiology, Department of Medicine, New York University School of Medicine, New York, New York; s Division of Cardiovascular Medicine, Department of Internal Medicine, Vanderbilt University Medical Center, Nashville, Tennessee; t Department of Biomedical Engineering, Vanderbilt University, Nashville, Tennessee; u Coroventis Research AB, Uppsala, Sweden; v Division of Cardiovascular Medicine, Department of Medicine, Stanford University, Stanford, California; w NTT Medical Center Tokyo, Tokyo, Japan; x Abbott Vascular, Santa Clara, California; y St Francis Hospital and Heart Center, Roslyn, New York

**Keywords:** CATH CMD, coronary flow reserve, coronary microvascular dysfunction, coronary microvascular function, index of microvascular dysfunction

## Abstract

Coronary microvascular dysfunction (CMD) can cause myocardial ischemia in patients presenting with angina without obstructive coronary artery disease (ANOCA). Evaluating for CMD by using the thermodilution technique offers a widely accessible means of assessing microvascular resistance. Through this technique, 2 validated indices, namely coronary flow reserve and the index of microcirculatory resistance, can be computed, facilitating investigation of the coronary microcirculation. The index of microcirculatory resistance specifically estimates minimum achievable microvascular resistance within the coronary microcirculation. We aim to review the bolus thermodilution method, outlining the fundamental steps for conducting measurements and introducing an algorithmic approach (CATH CMD) to systematically evaluate the coronary microcirculation. Embracing a standardized approach, exemplified by the CATH CMD algorithm, will facilitate adoption of this technique and streamline the diagnosis of CMD.

## Introduction

Coronary microvascular dysfunction (CMD) can cause ischemia in patients presenting with angina without obstructive coronary artery disease (ANOCA).^1^ Observational studies have revealed that up to half of the patients with ANOCA exhibit CMD, with a higher prevalence observed among women.^2^ The presence of CMD has been linked to a worse prognosis compared to individuals with normal microcirculatory function.^3^ Although the precise pathophysiological mechanisms underlying CMD remain under investigation, promising advancements have been achieved in the invasive assessment of this condition. The CORMICA (CORonary MICrovascular Angina) study showed that an invasive diagnostic evaluation using thermodilution in patients without obstructive CAD linked with medical therapy improved angina in patients with no obstructive CAD.^4^

The thermodilution technique offers a widely accessible means of assessing microvascular resistance. Similar to measuring cardiac output with the Swan-Ganz catheter, this method involves the use of an intracoronary wire equipped with temperature sensors to determine coronary flow by thermodilution of an indicator fluid.^5^ The intracoronary wire is also equipped with a distal coronary pressure sensor. Through this technique, 2 validated indices, namely coronary flow reserve (CFR) and the index of microcirculatory resistance (IMR), can be computed, both playing vital roles in investigating the coronary microcirculation. CFR quantifies the increase in blood flow from resting to hyperemic conditions across the entire coronary circulation, encompassing both the epicardial compartment and the microcirculation. In contrast, IMR estimates hyperemic minimal resistance of the coronary microcirculation.^6^^,^^7^

In this manuscript, we detail the bolus thermodilution method comprehensively and outline the fundamental steps and setup for conducting measurements, introducing an algorithmic approach to systematically evaluate the coronary microcirculation.

## Bolus thermodilution: Theoretical framework and clinical validation

The bolus thermodilution technique provides an estimate of flow by leveraging the indicator-dilution theory. In this approach, a bolus of an indicator liquid is injected into the proximal coronary circulation and the temperature is registered on the shaft of the coronary wire. As the indicator (saline) mixes with the blood and travels down the coronary circulation, its temperature is also measured using a distal temperature sensor. By analyzing the resulting dilution curves, which depict the change in temperature over time, the transit time (Tmn) of the indicator can be determined. The start of Tmn (t = 0) is defined as the time halfway through the injection, indicated by the temperature change at the ostium of the catheter. The end time is based on the temperature curve registered by the distal sensor. Subsequently, by dividing the volume by the transit time, the flow in mL/s can be reliably estimated. This method has proven to be effective in providing valuable insights into blood flow dynamics and is used in various clinical and research settings.^5^^,^^8^

De Bruyne et al^8^ and Pijls et al^6^ conducted pivotal studies to validate the bolus thermodilution technique for assessing coronary blood flow. These initial investigations revealed a close correlation between Tmn (transit time) and absolute flow measurements obtained using an external Doppler probe. Additionally, the calculation of CFR as the ratio of resting to hyperemic Tmn demonstrated a robust correlation with CFR derived from flow velocities measured by a Doppler wire.^6^ These findings showed the potential utility of the bolus thermodilution method for assessing coronary blood flow changes.^3^ It is important to highlight that CFR stands out as one of the most extensively validated metrics related to the coronary circulation, and its clinical significance spans across various scenarios, including patients with nonobstructive coronary artery disease. Numerous studies have consistently demonstrated the strong association between CFR and major cardiovascular events.^3^

Analogous to Ohm's law for electric circuits, the microvascular bed's resistance (R) can be represented as the ratio of the pressure gradient across a specific vascular territory (ΔP; in mm Hg) to the blood flow traversing this territory (Q; in mL/min). In this context, ΔP signifies the difference between the distal pressure in the coronary (Pd) and the coronary venous pressure (Pv). As Pv is typically considered negligible compared to Pd, the microvascular resistance equation can be succinctly expressed as follows:

$$Microvascular\;Resistance=\frac{Pd}{Flow}$$

As earlier demonstrated by De Bruyne et al,^8^ flow can be effectively estimated using bolus thermodilution as 1/Tmn. It is essential to highlight that flow is expressed in milliliters per minute, representing the ratio of volume to time; however, during thermodilution assessment, the vascular volume remains unmeasured. Fearon et al^7^ made a contribution by introducing the IMR as a novel application of bolus thermodilution. The formula for the IMR can be expressed as follows:

IMR=Pd(mmHg)xTmn

The same formula can be applied to resting conditions allowing us to assess the baseline resistance index.^9^ IMR, is, therefore, a metric surrogate of minimal microvascular resistance.

Since its introduction, the IMR has garnered validation across various clinical scenarios. In patients presenting with acute myocardial infarction, IMR has shown significant associations with indicators of microvascular function, including microvascular obstruction as assessed by magnetic resonance imaging, infarct size, and mortality.^3^^,^^10^^,^^11^

In stable patients undergoing percutaneous coronary intervention, IMR also has proven clinical relevance. Studies have demonstrated an association between IMR and peri-procedural myocardial infarction, subsequent myocardial infarction as well as cardiac death.^12^, ^13^, ^14^ IMR has demonstrated its usefulness as a prognostic marker in patients with cardiomyopathies and posttransplant settings.^15^^,^^16^ Nevertheless, it is relevant to recognize that an isolated increase of IMR with normal CFR does not portend an increased risk of events and that, in contrast to CFR, there is limited prognostic value of IMR in ANOCA patients.^3^^,^^17^^,^^18^

## Invasive procedure

A dedicated wire that measures pressure and temperature is required to evaluate the coronary microcirculation invasively using the bolus thermodilution technique (PressureWire X, Abbott Vascular). The shaft of the wire, on which the temperature-dependent electrical resistance is monitored, acts as a proximal thermistor, which allows for the detection of the start of the injections. A microsensor is mounted 3 cm from the tip at the transition of the radiopaque part of the wire and acts as a distal sensor enabling simultaneous recording of high-fidelity coronary pressure measurement as well as temperature measurement, with an accuracy of 0.02 °C. The proximal temperature sensor allows recording the time the saline bolus takes from the exit of the guide catheter until the distal coronary segment where the distal sensor is located. The bolus thermodilution injections are processed online using dedicated software (CoroFlow, Coroventis Research AB).

The mean transit times (Tmn) are derived from 3 brisk 3 mL saline injections, which are averaged, and the mean of the 3 values is used to calculate CFR and/or IMR. When injecting saline at room temperature through the guide catheter, a brisk injection leads to time-shifted, U-shaped temperature response curves in the proximal and distal temperature sensors. On occasion, due to a variety of factors discussed in the troubleshooting section, the 3 recordings may not always closely pair. In this case, outlier recordings may be easily corrected by repeating the injections in the same conditions. Because the quality of the thermodilution curves depends on the hand injection technique, we recommend using a 3 mL Luer Lock syringe attached to the manifold or autoinjector 3-way stop-cock. For CFR, the 3 Tmn are obtained in resting and hyperemic conditions. In the case of IMR, only Tmn obtained during hyperemic conditions is used for the calculation. For reliable CFR and IMR measurements, it is essential to have a steady hyperemic state.

## CATH CMD algorithm

To systematize bolus thermodilution measurements, we recommend following the CATH CMD algorithm (Central Illustration). This mnemonic aims at structuring the procedure while providing tips and tricks to acquire high-quality curves and correct results. The preparation of the pressure-temperature wire follows the same procedure as for fractional flow reserve (FFR) measurements with equalization of the pressure at the tip of the guide catheter before advancement into the distal segment of the coronary artery.

**Central Illustration:**
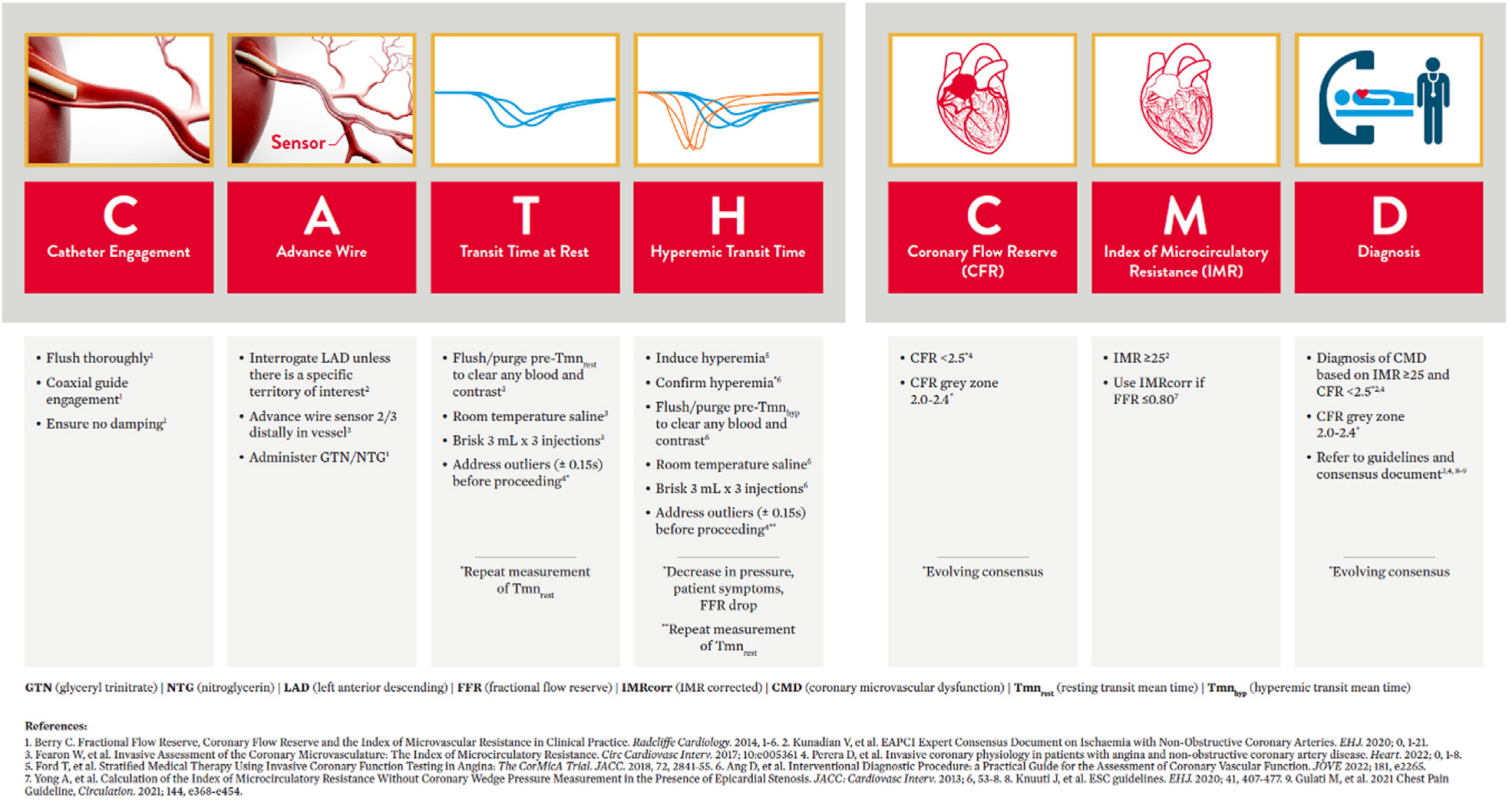
CATH CMD algorithm.

### Catheter engagement

Coaxial catheter engagement is a key factor in obtaining optimal temperature curves. Lack of coaxiality results in a spill into the aorta, resulting in insufficient saline reaching the thermistor located in the distal segment of the vessel and erroneous elevations in measurements of Tmn. Extra backup guide catheters can improve catheter engagement for thermodilution measurements, and constant assessment of the morphology of the aortic pressure curve to ensure the presence of a dicrotic notch and avoidance of pressure curve ventricularization is critical.

### Advance wire

In view of its larger subtended left ventricular mass and the fact that the majority of existing coronary physiology data in ANOCA were obtained from within this vessel, we advise interrogating the left anterior descending (LAD) artery unless anatomical scenarios hinder the measurement (eg, severe vessel tortuosity, small vessel, etc.). Of note, CMD may, however, develop in other territories (left circumflex, right coronary artery), and interrogation of the LAD artery is not necessarily representative of other circulations.^19^ Systemic anticoagulation is recommended before instrumentation of the coronary artery with a guide wire with control of activated clotting time. Before the wire is advanced, epicardial vasodilation with intracoronary nitrate administration is mandatory. The wire should be advanced, and the sensor should be placed in the transition between the mid and distal segments of the vessel, two-thirds along the vessel length (at least 6 cm down the vessel). Too proximal or distal a position affects the transit times and may influence the measurements. Subsequently, the proximal and distal thermistors are “equalized” by clicking “Zero Temperature” in the CoroFlow console.

### Transit time at rest

**Figure 1:**
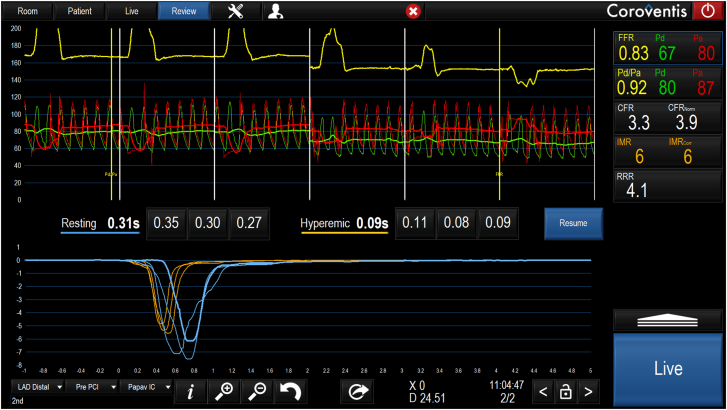
**Output after bolus thermodilution assessment in resting and hyperemic conditions.** Several pressure and thermodilution-based indexes will be displayed on the right panel of the Coroflow screen. The values are linked with the positioning of 2 yellow cursors (“Pd/Pa” and fractional flow reserve [“FFR”]) along the pressure recording, which is divided into 6 panels by white lines; the 3 on the left-hand side show the pressure during the 3 injections in resting conditions, and the 3 on the right obtained during hyperemic conditions. The Pd/Pa and FFR are shown from top to bottom. The coronary flow reserve (CFR) and index of microcirculatory resistance (IMR) follow these; both CFR and IMR values show their corrected values as CFR_norm_ and IMR_corr_. A CFR <2.5 is considered abnormal; values between 2.0 and 2.4 are considered to fall in the gray zone. An IMR >25 is considered indicative of high microvascular resistance. In cases of hemodynamically significant disease (FFR ≤0.80), it is advisable to use the IMR value corrected by FFR. This method is derived from the formula: Pa × Tmn × ([1.35 × Pd/Pa] – 0.32) and enables assessment of coronary microcirculatory in the presence of epicardial stenosis and collateral flow.^20^ The resistive reserve ratio (RRR) indicates the number of times that the index of resistance drops, and it is calculated as the ratio of basal resistance index and IMR. RRR has been shown to correlate with clinical outcomes in patients presenting with acute coronary syndromes.^9^ Finally, the value is also shown normalized by FFR as CFR_norm_.

Before starting bolus thermodilution measurements, it is recommended to flush with saline to clear blood and contrast from the system and the coronary artery. After clicking on “Start,” the first bolus thermodilution injection can be performed as indicated by the system with the message “Inject now.” While performing the injection, it is useful to assess the shapes of the temperature curves and compare them visually. Outliers should be repeated (see below for definition). The quantification of the transit times will appear automatically on the screen after each injection, and at the end of the 3 injections, the average of the 3 injections will also appear automatically on the screen (Figure 1).^9^^,^^20^

### Hyperemic transit times

After the 3 resting injections have been completed, the system will instruct to induce hyperemia, generally performed pharmacologically with adenosine via continuous infusion, although other approaches, including papaverine, nicorandil, nicardipine, regadenoson, or exercise, are also possible (Table 1). Hyperemia can be confirmed by a mild decrease in mean aortic pressure and a decrease in Pd/Pa or, in cases where intravenous adenosine is used, may also be identified by the patient’s symptoms, such as shortness of breath, hot flashes, or flushing. Once hyperemia is achieved, flush with saline again to clear any blood and contrast from the system. During hyperemia, 3 new 3 mL injections are performed to assess hyperemic flow. As with the resting injections, the quantification of transit times will appear automatically on the screen after each injection, and at the end of the 3 injections, the average of the 3 injections will be displayed next to the individual values. The injection of saline in the resting condition may itself elicit some hyperemia. Thus, it is important to wait 20 to 30 seconds between each resting injection to allow this effect to level off.

**Table 1:** Commonly used hyperemic agents.

Drug	Dose	Hyperemia duration	Half-life	Side effects	Advantages	Disadvantages
Adenosine IV	140 μg/kg/min	1-2 min	<10 s	• AV block • Chest tightness • Bronchospasm • Hypotension	• Steady-state hyperemia • Ostial lesion assessment possible • Pullback assessment possible	• Vasalva or deep respiration can interrupt drug infusion
Adenosine IC	36-120 μg LCA 18-60 μg RCA	3-8 s	<10 s	• AV block	• Well tolerated. • Repeat measurements can be performed quickly	• Incremental doses often required to ensure hyperemia • Pullback not feasible • Ostial lesion assessment not feasible
Papaverine IC	10 mg LCA 15 mg RCA	30-60 s	2 min	• QT prolongation • Ventricular tachycardia • Long half-life • Hypotension	• Can be used in patients with asthma and COPD • Pullback assessment possible	• Safety profile not as favorable as adenosine
Nicorandil IC	2 mg LCA 2 mg RCA	30 ± 15 s	6 min	• Hypotension	• Well tolerated (no AV block) • Can be used in patients with asthma and COPD • Pullback assessment possible	• Short plateau duration (IMR and pullback feasible only for experienced operators) • Not available in all countries • Need to stop sulfonylurea oral hypoglycemic agent 24 h before
Regadenoson IV	400 μg bolus	Variable (10 s to 10 min) Mean 163 s	2-4 min	• Chest discomfort • Bronchospasm • Hypotension	• Feasible in peripheral venous access • Ostial lesion assessment possible	• Hyperemia duration highly variable and might be not sufficient for pullback or IMR recording

AV, atrioventricular; COPD, chronic obstructive pulmonary disease; IC, introcoronary; IMR, index of microvascular resistance; IV, intravenous; LCA, left coronary artery; RCA, right coronary artery.

### CFR

An abnormal CFR (<2.5) in patients with nonobstructive CAD (FFR > 0.80) suggests the presence of impaired vasodilatory reserve and CMD.^1^ Bolus thermodilution may slightly overestimate the CFR value compared to Doppler measures of CFR.^21^^,^^22^ Therefore, CFR values between 2.0 and 2.5 should be considered to fall in the gray zone, and clinical judgment should be used in the interpretation of the findings.

### Microcirculatory resistance

IMR represents the minimal microvascular resistance, calculated as the distal coronary pressure multiplied by the hyperemic transit time. The current consensus recommends a cutoff of 25 IMR units to define CMD with high microvascular resistance in stable patients without coronary artery disease.^7^ In the presence of epicardial stenosis with FFR <0.80, the corrected IMR (IMR_c_), taking into account collateral flow, is displayed next to the IMR value and should be used.

### Diagnosis

The etiology of CMD is multifactorial and remains the subject of ongoing research. Endothelial dysfunction, inflammation, and alterations in the autonomic nervous system are all relevant pathophysiological mechanisms. Reduced nitric oxide bioavailability and increased oxidative stress contribute to endothelial dysfunction, leading to impaired vasodilation and microvascular constriction.^23^ Additionally, inflammatory processes within the coronary microvasculature may further compromise blood flow regulation in specific clinical scenarios. The dysregulation of the autonomic nervous system, characterized by sympathetic overactivity and parasympathetic withdrawal, may also play a role in CMD pathogenesis. Furthermore, heart failure, systemic factors, including raised left ventricular end-diastolic pressure (LVEDP), microvascular rarefaction or compression, platelet plugging, and distal embolization of thrombus in the setting of myocardial infarction, are important structural noncoronary contributors to elevated microvascular resistance.^24^ Understanding these mechanisms is vital for the development of targeted therapeutic interventions.

**Figure 2:**
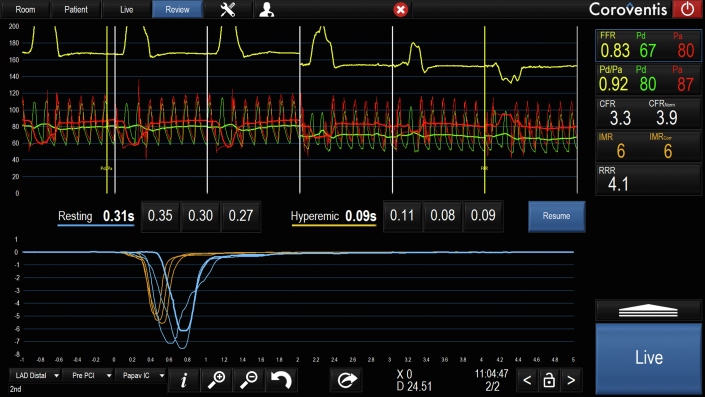
**Case examples of patients with normal and abnormal microvascular function.** (**A**) shows a case of normal microvascular function with normal coronary flow reserve (CFR), index of microcirculatory resistance (IMR), and fractional flow reserve (FFR). (**B**) shows a case of borderline FFR (0.80), low CFR, and normal IMR; this is an example of functional coronary microvascular dysfunction. (**C**) shows a case of low CFR driven by high microvascular resistance (IMR of 37); this is an example of structural coronary microvascular dysfunction. (**D**) shows a case of normal CFR but high IMR.

In clinical practice, low CFR can arise from 2 distinct mechanisms: firstly, from increased resting coronary artery blood flow, indicating reduced microvascular resistance at rest; and secondly, from reduced hyperemic flow due to elevated hyperemic microvascular resistance.^25^ These 2 scenarios give rise to different outcomes when using CFR and IMR as a diagnostic tool. In cases where low CFR results from increased resting blood flow (short resting Tmn), IMR will typically appear normal. Conversely, when low CFR results from prolonged hyperemic transit time, leading to elevated IMR values (IMR > 25), it indicates abnormal microvascular function. Case examples of different scenarios with bolus thermodilution are shown in Figure 2.

**Figure 3:**
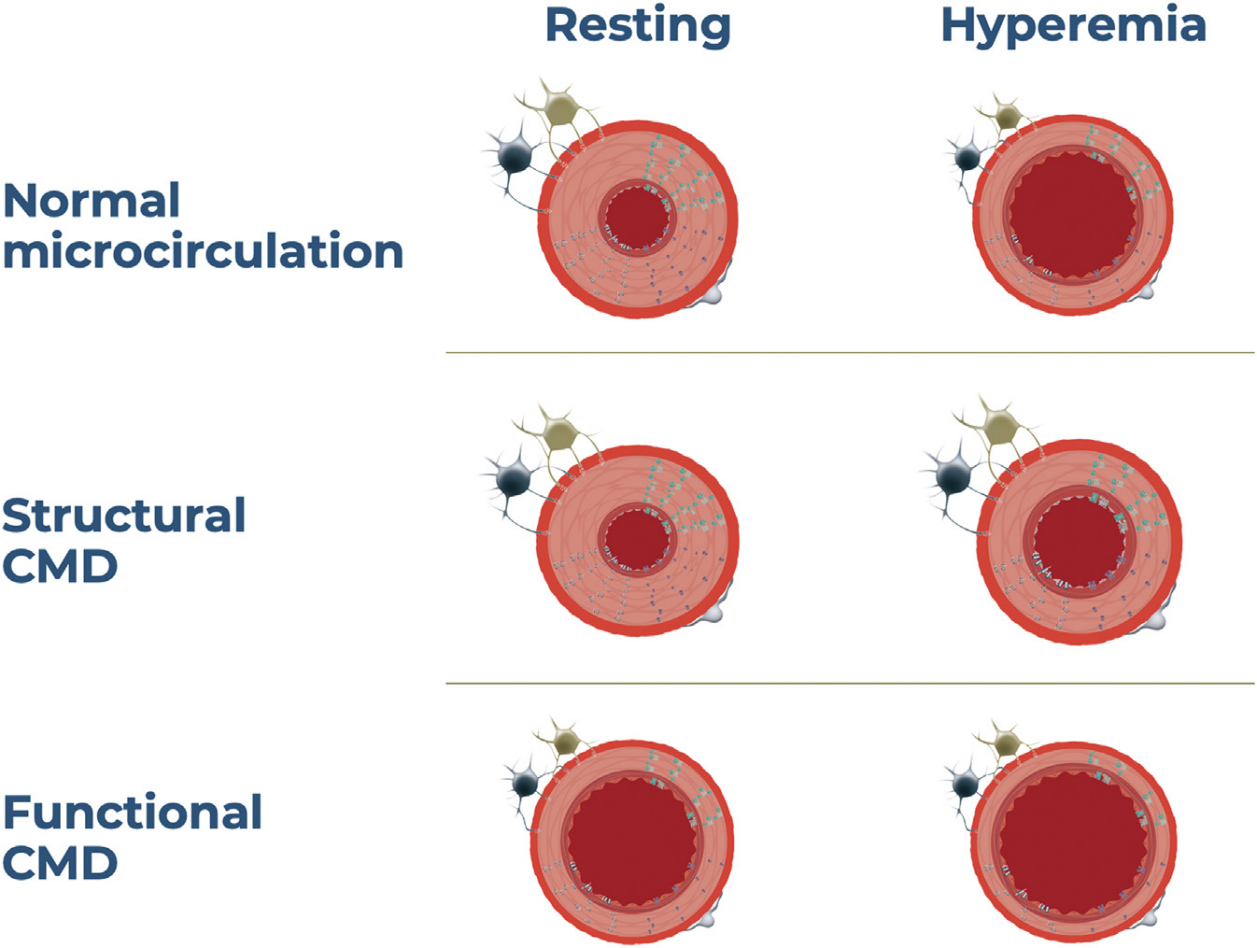
**Coronary microvascular dysfunction (CMD) endotypes**.

Evolving consensus suggests that these 2 distinct presentations represent separate entities of CMD, classified as functional and structural CMD, respectively (Figure 3). Patients with low CFR due to short Tmn at rest can be identified as having functional CMD, whereas those with low CFR resulting from prolonged hyperemic Tmn and high IMR are classified as having structural CMD. The clinical implications and long-term prognosis of these separate CMD subtypes are the subject of intense investigation. A specific scenario that deserves to be mentioned is normal CFR (>2.5) with high IMR. In these cases, it becomes crucial to recognize that both resting and hyperemic transit times are considerably prolonged. This clinical presentation suggests that the microvascular bed possesses sufficient vasodilator capacity to increase coronary flow by more than 2.5 times during hyperemia. However, despite this capability, the hyperemic flow remains lower than expected due to persistently elevated microvascular resistance. This clinical scenario appears not to pose additional risk to patients.^17^

The therapeutic approach to patients with CMD is a field of active investigation, and pharmacological therapies are principally directed at reducing oxygen demand with beta-blockers and calcium channel antagonists. However, the EDIT-CMD trial (Efficacy of Diltiazem to Improve Coronary Microvascular Dysfunction) showed no benefit of diltiazem, when compared with a placebo, in patients with CMD. However, diltiazem reduced the prevalence of epicardial spasms.^26^ More recently, the ChaMP-CMD (Characterizing Mechanisms in Patients with Coronary Microvascular Disease to Stratify Therapy) study demonstrated the clinical benefit of amlodipine and ranolazine in patients with CMD with a documented improvement in exercise capacity.^27^ The potential treatment strategies for CMD are beyond the scope of this manuscript.^28^

## Tips, tricks, and troubleshooting

Patient preparation plays a pivotal role in ensuring an accurate assessment of the coronary circulation; however, considerable operator variation exists. We recommend withholding vaso-active drugs, such as calcium channel blockers and calcium antagonists, for 24 to 48 hours before the procedure. Additionally, as with any hyperemic physiological assessment, patients should be advised to abstain from consuming caffeine and its derivatives on the day prior to the assessment, particularly in cases where adenosine is utilized as the hyperemic agent.

Coronary microvascular testing is optimally done as part of more comprehensive invasive testing, which includes acetylcholine infusion for endothelial function assessment and provocation test for coronary vasospasm. In situations where a concomitant test for the evaluation of endothelial dysfunction or vasospastic angina with acetylcholine is planned, consideration should be given to the potential impact of upfront use of vasodilator cocktails, which are commonly employed to minimize radial spasms. The order of the examination remains a matter of debate, some centers advocate for acetylcholine testing first, without prior nitrates and vessel instrumentation, followed by nonendothelial-dependent evaluation. There is ample experience using radial access with 6 French guides for comprehensive invasive physiologic testing. In some situations, such as the presence of radial spasm, 5 French guides may be considered; however, this may increase the errors detected by the software during bolus thermodilution injections.

To obtain accurate physiological measurements, stability of hemodynamic conditions is critical, as the coronary circulation autoregulates in response to changes in blood pressure. These mechanisms can impact the evaluation of coronary flow and microvascular resistance. When assessing resting blood flow, it becomes essential for the patient's conditions to closely resemble a true resting state. However, achieving this can be challenging, as many patients may experience heightened distress during invasive procedures. Additionally, specific factors such as pain at the arterial access site can further deviate from a genuine resting state. To mitigate these challenges, it is advisable to conduct measurements in a quiet environment. Conscious sedation can be considered. However, when this is not possible, it is important to account for these factors when interpreting the results, particularly in supposedly “resting” conditions. Elevated LVEDP increases mechanical compression of the subendocardial layer and is associated with CMD, impairments in cardiac function, and heart failure with preserved ejection fraction. LVEDP is also prognostically important.^29^ For these reasons, measuring LVEDP complements the assessment of coronary microvascular function in the catheter laboratory and should be integrated into the interpretation of the resistance measurements. There are several potential situations during the procedure where the CoroFlow system can detect errors related to the technique. The most frequent errors and corrective actions are shown in Table 2. One of the most frequently encountered situations is variability between Tmn. Variability is physiological, as coronary blood flow changes rapidly between systole and diastole. Hence, the need for an average of measurements, and also for a resting state environment. The CoroFlow software will identify this by displaying a yellow box around the transit times. Any transit time value deviating by more than 25% from the average value will be flagged automatically by the system with a yellow box around the Tmn. The system employs an automatic adjustment of the threshold for detecting high variability in Tmn based on the average Tmn value. For average Tmn values greater than or equal to 0.25, a cutoff of 30% variability was utilized, whereas in cases where Tmn was less than 0.25, a threshold for maximal variability of 50% was adopted. We recommend replacing these values by clicking on the outlying value and performing an extra injection. It should be noted that injection of saline in the resting state itself can introduce hyperemia.^30^ Thus, subsequent resting Tmn may be artificially too short. A time interval of 20 to 30 seconds between resting injections is therefore advised. After the third saline injection, the recording is saved, and the results are provided for clinical interpretation.

**Table 2:** Potential errors during bolus thermodilution measurements.

Type of error	Reason	Corrective actions
Slow inject	Injection too slow with >0.60 s between the start of the decline in temperature and the nadir at the proximal thermistor	• Use a 3 cc syringe • Inject with a rapid and vigorous motion • Make certain the injection is even with a distinct end
Timeout	Temperature did not return to baseline in time (> 8 s) at the distal thermistor	• Check that the guide is not too deeply engaged • Confirm that there is flow in the artery, if too occlusive stenosis the case may be unsuitable for thermodilution • Make sure that baseline temperature is 0 • Zero temperature and retry injection
Low amp	The temperature response did not reach the minimum limit during injection (<–1 °C) at the distal thermistor	• Verify guide engagement • Flush guide with fresh room temperature saline • Increase bolus or use slightly chilled saline if needed • Make sure wire is not too distal in vessel • Zero temperature and repeat injection

Finally, it is very important to identify and record the vessel assessed, the phase of the procedure, and the hyperemic agent used in all recordings. This is facilitated in the CoroFlow platform with specific fields in the bottom left corner of the screen.

## Current limitations and future perspective

Knowledge on the diagnosis, clinical implication, and management of CMD is rapidly evolving, but significant uncertainties persist. In this document, we advocate for the systematic assessment of the LAD artery; however, the potential added clinical value of multivessel interrogation remains a question for ongoing research. Studies have shown that CFR derived from bolus thermodilution may differ from that obtained through Doppler or continuous thermodilution measurements.^22^ Furthermore, bolus thermodilution-derived parameters can vary by up to 20% with repeated measurements.^31^ Therefore, it is essential to use the appropriate technique, emphasizing the rationale for standardizing this procedure, as outlined in the present document.

Although IMR has been proposed as a metric specific to the coronary microcirculation, it has been shown to depend on the subtended myocardial mass, which is strongly related to vessel volume.^32^ Given the assumptions embedded in the IMR calculation, more research is needed to understand the influence of mass and vessel volume on IMR and better the clinical implications of this finding. Several prospective studies are ongoing to increase our understanding of how to diagnose and manage CMD.

## Conclusion

Bolus thermodilution represents a reliable and efficient approach to assessing the coronary microcirculation. Thus, embracing a standardized approach, exemplified by the CATH CMD algorithm, will facilitate the widespread adoption of this technique and streamline high-quality diagnosis of CMD in patients with nonobstructive coronary artery disease. The increasing awareness of CMD and standardization of diagnostic tools have the potential to improve the outcomes in patients with nonobstructive coronary artery disease.
